# Adherence with urate-lowering therapies for the treatment of gout

**DOI:** 10.1186/ar2659

**Published:** 2009-03-27

**Authors:** Leslie R Harrold, Susan E Andrade, Becky A Briesacher, Marsha A Raebel, Hassan Fouayzi, Robert A Yood, Ira S Ockene

**Affiliations:** 1 Department of Medicine, the Meyers Primary Care Institute, 425 North Lake Avenue Worcester, MA 01605, USA; 2 Department of Medicine, University of Massachusetts Medical School, 377 Plantation Street Worcester, MA 01605, USA; 3 The HMO Research Network Center for Education and Research in Therapeutics, Department of Medicine, University of Massachusetts Medical School, 377 Plantation Street Worcester, MA 01605, USA; 4 Department of Research, Kaiser Permanente, Colorado Institute for Health Research, PO Box 378066 Denver, CO 80237-8066, USA; 5 Department of Medicine, Fallon Clinic, 425 North Lake Avenue Worcester, MA 01605, USA

## Abstract

**Introduction:**

Adherence to urate-lowering drugs (ULDs) has not been well evaluated among those with gout. Our aim was to assess the level and determinants of non-adherence with ULDs prescribed for gout.

**Methods:**

We identified persons using two integrated delivery systems aged 18 years or older with a diagnosis of gout who initiated use of allopurinol, probenecid or sulfinpyrazone from 1 January 2000 to 30 June 2006. Non-adherence was measured using the medication possession ratio (MPR) over the first year of therapy and defined as an MPR < 0.8. Descriptive statistics were calculated and logistic regression was used to estimate the strength of the association between patient characteristics and non-adherence.

**Results:**

A total of 4,166 gout patients initiated ULDs; 97% received allopurinol. Median MPR for any ULD use was 0.68 (interquartile range (IQR) 0.64). Over half of the patients (56%) were non-adherent (MPR < 0.8). In adjusted analyses, predictors of poor adherence included younger age (odds ratio (OR) 2.43, 95% confidence interval (CI) 1.86 to 3.18 for ages <45 and OR 1.44, 95% CI 1.08 to 1.93 for ages 45 to 49), fewer comorbid conditions (OR 1.46, 95% CI 1.20 to 1.77), no provider visits for gout prior to urate-lowering drug initiation (OR 1.28, 95% CI 1.05 to 1.55), and use of non-steroidal anti-inflammatory drugs in the year prior to urate-lowering drug initiation (OR 1.15, 95% CI 1.00 to 1.31).

**Conclusions:**

Non-adherence amongst gout patients initiating ULDs is exceedingly common, particularly in younger patients with less comorbidity and no provider visits for gout prior to ULD initiation. Providers should be aware of the magnitude of non-adherence with ULDs.

## Introduction

Gout is a common inflammatory arthropathy affecting up to 5 million Americans, and yet little is known regarding the dynamics of chronic medication use in these patients [1]. Both recurrent and tophaceous gout require sustained treatment with urate-lowering drugs (ULDs) to reduce the frequency of acute gouty attacks and prevent urate nephropathy, uric acid nephrolithiasis, and the deposition of tophi: a common cause of progressive joint damage, deformity and functional impairment [2,3]. Chronic use of ULDs is necessary for lowering and maintaining serum uric acid levels to a target threshold of <6.0 mg/dl, as this is associated with fewer gout flares, reduction of tophus area, and depletion of urate crystal stores in synovial tissues [4-7].

Surprisingly little is known about how patients with gout manage their ULD therapy even though adherence is critical for preventing the painful and damaging effects of the disease [8,9]. A recent review of the literature detected very few studies on this topic [10-13]. In addition, the studies varied in terms of study design, study populations, and definitions of adherence. A recent study comparing medication non-adherence across seven common chronic conditions such as hypertension, osteoporosis and diabetes mellitus found adherence was lowest with gout [8]. Interestingly, predictors of adherence varied by condition. For example, younger age was associated with lower adherence in the treatment of hypothyroidism and diabetes but not in seizure disorders. Additionally, as comorbidity burden increased, adherence to osteoporosis treatment decreased whereas adherence to medications used to treat hypertension and hypercholesterolemia increased.

While non-adherence in certain other chronic conditions (for example, hyperlipidemia) may not be associated with short-term consequences, interruptions in ULD use can trigger or prolong acute gout attacks [3,4]. The most recent publication looking at predictors of adherence in gout patients was focused on the elderly (mean age of the study population was 79), and the overwhelming majority of patients were women [13]. That study population is not representative of patients typically followed in an outpatient medical practice. Therefore, more investigation in the rates and causes of non-adherence in the treatment of gout is necessary, particularly in men and younger populations. Specifically, the objective of this study was to analyze the rates and predictors of non-adherence with ULDs in a representative sample of gout patients. We hypothesized that patients with more frequent gout attacks would be more adherent.

## Materials and methods

### Setting and dataset

Two health care delivery systems involved in the Health Maintenance Organizations (HMO) Research Network Center for Education and Research on Therapeutics (CERTs) participated in this study [14]. The two systems are located in the Northeast and Rocky Mountain regions of the USA, with a combined population of approximately 650,000 (2006 figures). We identified the study cohort using the Virtual Data Warehouse (VDW), a previously-developed mechanism used to produce comparable data across participating health plans [15]. The VDW uses standardized file definitions, uniform data dictionaries for each content area, and common formats for each data element. These common file structures enable analysts at each site to write programs to extract and/or analyze data at all participating sites. For this study, the analytic dataset included computerized information on utilization of health care services including membership, pharmacy dispensing data, and selected hospital and ambulatory diagnoses and procedures for all enrollees of the health plans from 1 January 1999 to 30 June 2007. Institutional review boards at each participating organization approved this study.

### Study population and design

We identified members from the dataset who had an International Disease Classification, version 9 (ICD-9) code for a gout diagnosis (codes 274.XX), were aged 18 years or older at the time of the first ULD dispensing, were dispensed a ULD (allopurinol, probenecid or sulfinpyrazone) between 1 January 2000 and 30 June 2006 and were continuously enrolled in the health plan with drug coverage during the period 12 months prior to and 12 months following the first ULD dispensing. Our analysis focused on new users of therapy, which was defined as no dispensing of a ULD in the prior 6 months.

### Adherence measure

We used the medication possession ratio (MPR) to measure adherence [16]. The MPR was calculated as the days supply of medication dispensed during the follow-up year divided by the number of days in the year and is a reliable measure of adherence [17]. The MPR was determined based on pharmacy dispensing records, including the number of days supplied. Use of the MPR allowed comparisons with other studies who used similar methods in examining adherence to ULDs [11-13]. As has been performed in other prior studies, the MPR was dichotomized at 80% in multivariable analyses, with an MPR of <80% considered non-adherence [11-13].

### Covariates and measures

Patient characteristics were assessed, including those covariates identified as potential correlates of adherence based on previous studies [11-13,18]. These included demographic factors (age and sex), health care utilization (visits to providers for gout both prior to and after ULD initiation, all provider visits prior to ULD initiation, and number of hospitalizations prior to ULD initiation), specific comorbidities, other medications used to treat symptomatic gout, and medications that can trigger gout. Age (as of first ULD dispensing) and sex were ascertained from the demographic data. We ascertained the presence of comorbidities from the ICD-9 diagnosis codes associated with ambulatory, emergency department, and inpatient care during the time period 12 months prior to and 12 months following the first ULD dispensing. Comorbidities of interest included coronary heart disease, diabetes mellitus, dyslipidemia, hypertension, nephrolithiasis, peripheral arterial disease, renal insufficiency and renal failure. A Charlson comorbidity score (the score does not include a gout diagnosis) was ascertained based on a range of diagnoses and procedures in the 12 months prior to the first ULD dispensing [19].

Medication usage was identified by determining dispensings using national drug codes (NDCs) for the following drug classes: diuretics (thiazide, potassium sparing, loop and others), both cyclo-oxygenase selective and non-selective non-steroidal anti-inflammatory drugs (NSAIDs), colchicine, uricosuric drugs, xanthine oxidase inhibitors (allopurinol) and glucocorticoids. Acute medications for gout were identified based on dispensings of NSAIDs, colchicine and glucocorticoids in the 1 to 12 months prior to the first ULD dispensing. Due to the recommendations that patients receive prophylactic medications prior to ULD initiation, we looked separately at dispensings of NSAIDs, colchicine and glucocorticoids in the 30 days prior to and including the date of the first ULD dispensing.

### Analyses

The characteristics of the study population were examined. The MPR was examined both by use of specific ULD agents and by use of any ULDs, so that if patients switched agents, they could still be considered adherent. Adherence using the MPR (≥80%) based on initiated ULD (allopurinol vs probenecid) was examined using the χ^2 ^test. We then assessed correlates of non-adherence with use of any ULD. Logistic regression was used to estimate the strength of the association between patient characteristics and non-adherence. Variables included in the models included age, gender, health care utilization (number of provider visits for gout, number of provider visits for any diagnosis and hospitalizations prior to ULD initiation), Charlson comorbidity score, use of diuretics, use of acute gout medications in the 1 to 12 months prior to ULD initiation (NSAIDs, colchicine and glucocorticoids), use of acute gout medications in the 30 days prior to and including the date of ULD initiation, and HMO. A backward elimination method was used to determine the variables to include in the multivariable models, including retaining all variables considered to be potentially important. Variables with *P *values < 0.10 remained in the final models. All analyses were run using SAS version 9.1 (SAS Institute, Cary, NC, USA).

## Results

A total of 4,166 patients with a diagnosis of gout who were initiated on a ULD during the study period. The mean age of the population was 62 (± 14) years and 75% were male (Table 1). In the 12 months prior to and including the date of ULD initiation, the mean number of provider visits for gout was 1.67 (± 1.62) with 288 (7%) patients having no in person encounters during that period. After ULD initiation, the mean number of provider visits for gout was 1.73 (± 2.43). Comorbidities were common, including hypertension (70%), dyslipidemia (47%), coronary heart disease (24%), and diabetes (24%). More than half of the patients were receiving diuretics.

**Table 1:** Baseline characteristics of patients initiating urate-lowering therapy

Characteristic	Total population (N = 4,166)
Mean age, years (SD)	62 (14)
Gender, N (% male)	3,113 (75)
Number of provider visits for gout prior to ULD initiation, mean (SD)*	1.67 (1.62)
Number of physician visits for any diagnosis prior to ULD initiation, mean (SD)*	6.16 (5.81)
Hospitalization, N (%)*	782 (19)
Number of provider visits for gout after ULD initiation, mean (SD)†	1.73 (2.43)
Charlson comorbidity score, mean (SD)	1.06 (1.64)
Associated comorbidities, N (%):	
Hypertension	2,904 (70)
Dyslipidemia	1,976 (47)
Coronary heart disease	1004 (24)
Diabetes mellitus	989 (24)
Renal insufficiency	816 (20)
Renal failure	629 (15)
Peripheral arterial disease	243 (6)
Nephrolithiasis	196 (5)
Medications associated with gout or difficulty treating gout, N (%):	
Thiazide diuretics	1,569 (38)
All diuretics	2,259 (54)

*Over the 12 months prior to initiation of a ULD; †over the 12 months after initiation of a ULD.

SD, standard deviation; ULD, urate-lowering drug.

**Figure 1:**
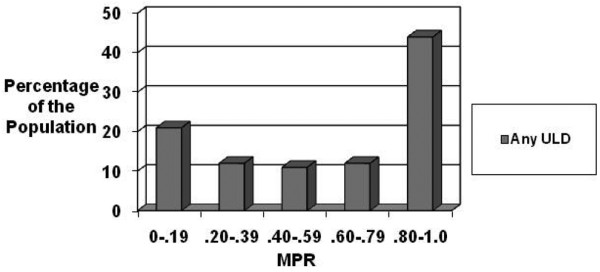
The distribution of the medication possession ratio (MPR) for any urate-lowering drug (ULD) use. The distribution of the study population in terms of MPR for any ULD use was examined.

The vast majority of patients initiated treatment with allopurinol (97%), with 3% receiving probenecid and less than 1% (only one patient) receiving sulfinpyrazone for urate lowering therapy (Table 2). The median MPR was 0.68 (interquartile range 0.64) for allopurinol and 0.49 (interquartile range 0.70) for probenecid. Adherence did not differ based on initiated ULD (allopurinol vs probenecid; *P *< 0.11) non-adherence (MPR < 0.8) to any ULD during the first year of therapy occurred in 56% of patients (Figure 1).

**Table 2:** Use of acute and chronic gout medication treatments in initiators of urate-lowering drugs (ULDs)

ULD	Population, N (%)
Allopurinol	4,042 (97)
Probenecid	123 (3)
Sulfinpyrazone	1 (0)
Acute gout treatments prior to ULD initiation*:	
NSAIDs	2,003 (48)
Colchicine	862 (21)
Glucocorticoids	742 (18)
Possible gout flare prophylaxis prior to ULD initiation†:	
NSAIDs	1,554 (37)
Colchicine	1,189 (29)
Glucocorticoids	675 (16)

Total population N = 4,166.

*Based on dispensings 1 to 12 months prior to ULD initiation; †based on dispensings 30 days prior to and including the date of ULD initiation.

NSAID, non-steroidal anti-inflammatory drug.

In the 1 to 12 months prior to ULD initiation, use of acute gout medications was common (48% of patients were treated with NSAIDs, 21% colchicine and 18% glucocorticoids). Use of acute gout medications in the 30 days prior to and including the date of ULD initiation (potentially dispensings for gout prophylaxis) occurred in the 64% of patients. NSAIDs and colchicine were used in 37% and 29% of patients within the 30 days prior to the date of ULD initiation, respectively. While glucocorticoids are not the typical medications used for gout prophylaxis, 16% of patients received these agents.

After controlling for health plan, in the adjusted analyses, factors that increased the probability of non-adherence included younger age (odds ratio (OR) 2.43, 95% confidence interval (CI) 1.86 to 3.18 for ages <45 and OR 1.44, 95% CI 1.08 to 1.93 for ages 45 to 49 compared with 75 and older), fewer comorbid conditions (OR 1.46, 95% CI 1.20 to 1.77 for Charlson score of 0 compared with a score of 3 or more), no provider visits for gout prior to ULD initiation (OR 1.28, 95% CI 1.05 to 1.55 for no visits compared with three or more visits), and use of NSAIDs in the 1 to 12 months prior to ULD initiation (OR 1.15, 95% CI 1.00 to 1.31) (Table 3). Those aged 55 to 64 were significantly more likely to be adherent. The number of physician visits prior to ULD initiation, hospitalizations and use of prophylactic medications prior to ULD initiation were not associated with non-adherence. As a sensitivity analysis, we re-ran the models excluding those with only one prescription for a ULD in case those patients were non-adherent due to an adverse reaction. The strengths of the associations were essentially the same.

**Table 3:** Adjusted logistic regression model for non-adherence (medication possession ratio (MPR) < 0.80)*

Variable†	Adjusted odds ratio (95% confidence interval)
Sociodemographic:	
Gender, female	1.03 (0.88 to 1.19)
Age	
<45	2.43 (1.86 to 3.18)
45 to 49	1.45 (1.08 to 1.93)
50 to 54	1.09 (0.86 to 1.39)
55 to 59	0.78 (0.61 to 0.99)
60 to 64	0.75 (0.59 to 0.96)
65 to 69	0.84 (0.67 to 1.04)
70 to 74	0.83 (0.67 to 1.02)
75 +	1.00
Health status:	
Charlson comorbidity score	
0	1.46 (1.20 to 1.77)
1	1.21 (0.98 to 1.51)
2	1.20 (0.94 to 1.53)
3 or more	1.00
Gout care:	
Number of provider visits for gout prior to ULD initiation	
0	1.28 (1.05 to 1.55)
1	1.09 (0.92 to 1.30)
2	1.00 (0.83 to 1.21)
3 or more	1.00
Use of acute gout meds 1 to 12 months prior to urate-lowering drug initiation	
NSAIDs	1.15 (1.00 to 1.31)

*A backward elimination method was used retaining all variables considered to be potentially important. Variables with *P *values < 0.10 remained in the final models. The Hosmer-Lemeshow goodness of fit had a *P *value of 0.70; †model after controlling for health plan.

NSAID, non-steroidal anti-inflammatory drug; ULD, urate-lowering drug.

## Discussion

In this study, we found poor adherence to newly-initiated ULDs was common in gout patients, with only 44% of the study population achieving an MPR > 0.80 in the first year of therapy. Predictors of non-adherence included age less than 50 years, fewer comorbid conditions based on the Charlson score, no provider visits specifically for gout care prior to ULD initiation and the use of NSAIDs in the year prior to ULD initiation. These findings suggest that younger and healthier patients with gout may be at particularly high risk for medication non-adherence, potentially due to a lack of experience in managing other chronic conditions or seeking care. In addition, these patients may have a knowledge deficit in how and when to use their medications. We are currently conducting in-depth patient interviews to explore these factors.

Our work is consistent with others showing adherence with ULDs is generally poor, ranging from 44% to 64% of the study populations [8,11-13]. Previous studies also confirm our finding that younger age is associated with non-adherence [8,11,13]. Interestingly adherence was best in gout patients in the middle age groups (ages 55 to 64). This finding has been seen in the medication management of other chronic conditions, such as diabetes [20].

This study adds to the body of knowledge regarding non-adherence in the management in gout. First of all, we evaluated adherence in new users of ULDs and included a sample of gout patients that reflects the typical outpatient medical practice. In addition, we evaluated measures of gout care that have not been previously examined including number of physician encounters for gout prior to ULD initiation. Lack of provider visits associated with a gout diagnosis prior to ULD initiation was associated with non-adherence. This suggests a face-to-face meeting focused on gout is needed to discuss the rationale and goals of medication treatment of gout. Interestingly, we found that 7% of our population had no encounters prior to initiating ULDs. We surmise this occurred when patients called the physician's office for a prescription before a visit could occur. Since these patients may not be new users but rather patients with a long gap since their last prescription, we conducted a subanalysis excluding those patients from the model. Non-adherence was still associated with younger age and fewer comorbid conditions.

Use of NSAIDs, a class of medications used to treat acute gout, prior to ULD initiation was also significantly associated with non-adherence and the relationship persisted even when we controlled for use of prophylactic medications prior to ULD initiation. This suggests that patients with likely inadequately controlled gout prior to ULD initiation (reflecting the need for prescription NSAIDs) are less adherent. This finding was contrary to our *a priori *hypothesis that active gout would be linked to higher adherence. Interestingly, lack of prophylactic medications (NSAIDs or colchicine dispensed in the 30 days prior to and including the date of ULD initiation) was not associated with non-adherence.

These results have practical implications for clinicians. While many providers assume patients with painful arthritic conditions are adherent to their medications, our work has shown that this is not the case. Providers and health care systems may wish to identify patients at high risk for non-adherence based on the factors identified in this study. In those high-risk subsets of the gout population, providers could emphasize the rationale and benefits of ULDs in the treatment of gout and the consequences of non-adherence. Of note, *post hoc *analyses revealed that fewer number of provider visits for gout following ULD initiation were also associated with greater non-adherence both in adjusted and non-adjusted analyses. In light of these data, providers may wish to automatically schedule follow-up encounters after ULD initiation to review clinical status and to encourage medication adherence.

One of the strengths of this study is the sample inclusion criterion of a diagnosis of gout, which other studies have not featured. While this does not eliminate misclassification bias, it does reduce the likelihood that patients were receiving ULDs for the treatment of asymptomatic hyperuricemia, in which treatment recommendations and goals may differ from the standard lifelong treatment of gout with ULDs [4,21]. We also included data from two health delivery systems in different geographic regions, which reduced the influence of regional prescribing practices on our results and increased generalizability. Additionally, we were able to explore the relationship between gout-related care, such as provider visits for gout, use of acute gout medications prior to ULD initiation and use of potentially prophylactic medications. Lastly, given the study population had pharmacy benefits and ULDs are relatively inexpensive, we minimized the likelihood that financial constraints influenced adherence.

This study has several limitations. We conducted a retrospective analysis of pharmacy dispensings to measure adherence. Therefore, we could not confirm whether patients actually took the medications that they had filled. In addition, we did not have access to the patients or the clinical records to ascertain the reasons for gaps in therapy. We could not determine whether patients became non-adherent due to adverse reactions, at the suggestion of their provider, or secondary to other factors. However, it does seem less likely that non-adherence was due to an adverse reaction since when we re-ran the analyses excluding those who filled only one prescription the strengths of the associations were essentially the same.

Additionally, due to the limitations of pharmacy dispensing records we were not able to confirm the indication for NSAIDs and colchicine dispensed within the 30 days prior to and including the date of the first ULD dispensing. Patients may have also obtained over the counter NSAIDs that would not be captured by this study. However, since all the patients had pharmacy benefits, there is a financial incentive to obtain a prescription NSAID rather than take numerous over the counter pills to replicate the prescription dosage.

We did not have access to laboratory results and thus were unable to assess the impact of ULD adherence on uric acid levels. We also did not have access to the specialty of the ULD prescriber and thus could not examine whether adherence was influenced by provider specialty, specifically whether patients cared for by rheumatologists were more adherent. In addition, we were unable to determine definitely if visits associated with a diagnosis of gout were for acute gout flares versus routine follow-up appointments. Therefore, we could not directly determine the relationship between adherence and the frequency of gout attacks. Lastly, the population was overwhelming Caucasian, and thus we could not examine whether patient race and ethnicity influenced adherence.

## Conclusions

Non-adherence with ULDs for the treatment of chronic gout occurred in the majority of the study population. While further research is needed to assess the clinical and economic consequences of non-adherence, it is likely the patients did not achieve the recommended reduction in serum urate levels and thus are at risk for future gout flares, nephrolithiasis and boney erosions. Providers should be aware of the magnitude of the problem of non-adherence with ULDs, and counsel patients on the need for ULD adherence.

## Abbreviations

CERTs: Center for Education and Research on Therapeutics; ICD-9: International Disease Classification; MPR: medication possession ratio; NDC: national drug codes; NSAID: non-steroidal anti-inflammatory drug; ULDs: urate-lowering drugs; VDW: Virtual Data Warehouse.

## Competing interests

The authors declare that they have no competing interests.

## Authors' contributions

All authors made substantive intellectual contributions to this study to qualify as authors. LH was involved in the conception and design of this project, analysis and interpretation of the data, and drafting of this article. SA was involved in the design of this project, data acquisition and analysis, interpretation of the data, and critiquing of drafts of this article. BB was involved in the design of this project, analysis and interpretation of the data, and critiquing of drafts of this article. MR was involved in data acquisition, interpretation of the data and critiquing of drafts of this article. HF was involved in the analysis of data from this study and interpretation and presentation of results. RY was involved in the interpretation of the data, and drafting of this article. IO was involved in the conception and design of this project, analysis and interpretation of the data, and critiquing of drafts of this article. All authors read and approved the final manuscript.
